# Early Empirical Anidulafungin Reduces the Prevalence of Invasive Candidiasis in Critically Ill Patients: A Case-control Study

**DOI:** 10.2478/jccm-2022-0006

**Published:** 2022-05-12

**Authors:** Md Jahidul Hasan, Sharmind Neelotpol, Raihan Rabbani

**Affiliations:** 1 Clinical Pharmacy Service, Department of Pharmacy, Square Hospitals Ltd, Dhaka, Bangladesh; 2 Department of Pharmacy, BRAC University, Dhaka, Bangladesh; 3 Internal Medicine and ICU, Square Hospitals Ltd., Dhaka, Bangladesh

**Keywords:** anidulafungin, critically ill patient, invasive candidiasis, Candida pneumonia, Candida urinary tract infection

## Abstract

**Introduction:**

Invasive candidiasis (IC) in critically ill patients is a serious infection with high rate of mortality. As an empirical therapy, like antibiotics, the use of antifungals is not common in intensive care units (ICUs) worldwide. The empirical use of echinocandins including anidulafungin is a recent trend.

**Aim of the study:**

The objective of this study was to assess the impact of empirical anidulafungin in the development of invasive candidiasis in critically ill patients in ICU.

**Methods:**

This retrospective case-control study was conducted on 149 patients with sepsis with/without septic shock and bacterial pneumonia. All the patients were divided into two groups. The ‘control group’ termed as ‘NEAT group’ received no empirical anidulafungin therapy and the ‘treated group’ termed as ‘EAT group’ received empirical anidulafungin therapy in early hospitalization hours.

**Results:**

Seventy-two and 77 patients were divided into the control and the treated group, respectively. Patients in EAT group showed less incidences of IC (5.19%) than that of the NEAT group (29.17%) (p = 0.001). Here, the relative risk (RR) was 0.175 (95% CI, 0.064-0.493) and the risk difference (RD) rate was 24% (95% CI, 12.36%-35.58%). The 30-day all-cause mortality rate in NEAT group was higher (19.44%) than that of in EAT group (10.39%) (p = 0.04). Within the first 10-ICU-day, patients in the EAT group left ICU in higher rate (62.34%) than that in the NEAT group (54.17%).

**Conclusion:**

Early empirical anidulafungin within 6 h of ICU admission reduced the risk of invasive candidiasis, 30-day all-cause mortality rate and increased ICU leaving rate within 10-day of ICU admission in critically ill patients.

## Introduction

Invasive candidiasis (IC) or acute disseminated candidiasis or disseminated *Candida* infection is a life-threatening fungal infection, mostly seen in the intensive care units (ICU) patients, characterized by the presence of *Candida* species in different organs of the living body, unlike common Candida infections in the mouth, throat and vagina [1]. Candidemia is characterized by the presence of infectious *Candida* species in the bloodstream, which is the most dangerous clinical manifestation of IC with high risk of disseminated *Candid*a infections in other parts of the body [2, 3, 4]. Being the fourth most common organism causing bloodstream infections, *Candida is responsible for 10% of all b*loodstream infections in hospitals with 42% to 63% of hospital mortality rate [5, 6]. Candida pneumonia (CP) is found mostly in critically ill patients as a consequence of disseminated *Candida* species-associated infection secondary to predisposing clinical conditions, such as overuse of antibiotics, hematologic malignancy, or immunosuppressive state of patients. Similarly, urinary tract infection (UTI) with *Candida* species, also known as *Candida* urinary tract infection (CUTI) or candiduria (detection of 10^5^ fungal CFU/mL of urine and other symptoms, including fever and suprapubic pain), is an infectious state, commonly occurred in critically ill patients having indwelling bladder catheter where *Candida* species are detected in urine either as colonization or contamination of specimens [3,4]. Like candidemia, both CP and CUTI are responsible for high rate of mortality and morbidity in ICU patients [1, 5, 6]. The candidemia, CP and CUTI are the most prevalent forms of fungal infection found among ICU patients, worldwide. This emergence of IC and candiduria in the critically ill patients with worrisome rate of hospital mortality, extended hospital length-of-stay and increased treatment cost is an alarm for the global human health.^1,2,5^ The violence of IC, lack of appropriate diagnostic facilities and often limitation of proper hospital IC management guidelines enhances the mortality rate in critical care areas [7, 8, 9]. Though critical areas are considered as the most privileged area for experiencing IC [1] but a recent 10-year long survey found the prevalence rate of IC in non-contaminated hospital samples was around 28% [8].

Evidence of empirical antifungal therapy in critically ill patients is limited and few conclusive studies do not strongly support this issue [5, 7, 10]. Over the last few decades, researchers found reduced rate of hospital mortality with adequate empirical antibacterial therapies. Few studies were conducted successfully to evaluate the outcomes of early initiating antifungal therapies in critically ill patients [11, 12, 13, 14]. Some studies illustrated that early appropriate antifungal therapies reduce the rate of mortality with improved clinical outcomes in critically ill patients [1, 13, 14]. Some experts gave importance on the use of appropriate antifungal therapies empirically in high-risk critically ill patients [13, 15, 16].

Reduced fluconazole susceptibility in the non-*albicans* isolates in multiple studies has switched the interest of clinicians from azoles to echinocandins and amphotericin B lipid formulations in the context of first-line antifungal therapy mostly, for the critically ill patients [17, 18, 19, 20]. Nowadays, preemptively, echinocandins are considered as the first drug of choice in most of the hospital-based IC guidelines in ICU [8]. Echinocandin antifungals were discovered in 1970s. As the first echinocandin, anidulafungin, a semisynthetic product of echinocandin B, was approved by the Food and Drug Administration (FDA) of the United States (US) in 2006 [21]. However, very limited clinical evidences on patient outcomes with empirical therapies in critically ill patients to date have highlighted the necessity of large control trials to address this issue clearly [5, 11, 20, 22]. The aim of this study was to evaluate the impact of early empirical therapy in the development of invasive candidiasis, length of ICU stay, and 30-day all-cause mortality in critically ill patients admitted in ICU with sepsis with or without septic shock and bacterial pneumonia.

## Methods

### Study design, participants and data collection

This retrospective case-control study was conducted in a tertiary level private hospital in Bangladesh. All the critically ill patients with the diagnosis of sepsis with or without septic shock and bacterial pneumonia admitted in the ICU of the hospital from July 2019 to December 2020 were included in the study. Clinical, therapeutic, demographic and microbiological histories of these patients were collected from that electronic database of the hospital. All the patients in the study had sepsis and confirmed bacterial pneumonia during their admission in the ICU, and septic shock was found in some patients and that was managed with proper medications within 24 h to 72 h of admission. Patients developed community-acquired bacterial pneumonia during their stay at home and this respiratory tract infection was considered as the primary source of sepsis. All the critically ill patients of the study received high-dose corticosteroids and broad spectrum antibiotics for the management of sepsis and bacterial pneumonia for at least two weeks, and the development of the secondary fungal infections in the patients may be due to the use of these drugs or for their compromised immune system. Only the first episodes of culture-proven invasive *Candida* species-associated infections were considered for this study. Here, infections with invasive *Candida* species were attributed as the isolation of *Candida* species from blood, tracheal aspirates/bronchoalveolar lavage and urine. Anidulafungin is a costly drug and was included in the ICU treatment protocol of the hospital. Anidulafungin was empirically given to patients who agreed to receive and provided written consent. The study segregated all the patients with sepsis with/without septic shock and bacterial pneumonia on the basis of receiving anidulafungin empirically or not. All the Patients in the treated group of the study received antidulafungin (ERAXIS™ (anidulafungin),100 mg lyophilized powder for injection, Pfizer Inc., United States) (200 mg×1 as loading dose; then 100 mg/ day intravenously for 14 days; intravenous administration technique: 100 mg of anidulafungin mixed with 130 mL of normal saline and infused over 90 min; 200 mg of anidulafungin mixed with 260 mL of normal saline and infused over 180 min) empirically within 6 h of ICU admission and considered in “Empirical Anidulafungin Therapy (EAT) group”. On the other hand, patients in the control group did not receive any empirical anidulafungin and considered in “No Empirical Antifungal Therapy (NEAT) group”. Patients of the NEAT group received only anidulafungin for the treatment of confirmed fungal infections after getting the confirmation of the presence of *Candida* species in the first microbiological culture report. The EAT group (treated) was compared with the NEAT group (control) in terms of all clinical outcomes and ICU leaving rate within 10-ICU-day. As per the ICU-protocol, repeat microbiological tests were collected in every 6 days interval.

### Diagnostic method

For the detection of *Candida* species (broadly identified as *Candida albicans*, or non-*albicans*) in biological samples (blood, tracheal aspirates or bronchoalveolar lavage, and urine) of study patients, specimens were sent to hospital microbiology laboratory after evaluating the patents for suspected fungal infection as per the hospital standard ICU protocol. Primarily, the sabouraud dextrose agar medium was used for the growth of fungi including *Candida albicans*, and non-*albicans*. Secondly, the susceptibility testing of isolated *Candida* species was confirmed by using an automated micro-broth dilution system, the BD Phoenix™ M50 (BD Life Sciences: Diagnostics, USA.). Based on microbiological reports, other underlying risk factors in critically ill patients (as per the ICU treatment protocol) were considered.

### Patient inclusion and exclusion criteria

Sample inclusion criteria:

- Patients with confirmed sepsis with or without septic shock and bacterial pneumonia confirmed by radiological examination (chest X-ray)
- Diabetes mellitus and /or hypertension as common comorbidity
- Patients received no antifungal therapy within the last 21 days of ICU admission
- No history of fungal infection within the last 3 months of ICU admission
- Received anidulafungin empirically within the first 6 h of ICU-admission (for the patients of EAT group); and received no empirical antifungal therapy (for the patients of NEAT group)

Sample exclusion criteria:

- Patients with the history of severe hepatic impairment, chronic kidney disease or end stage renal disease, any kind of elective surgery within 3 weeks of ICU admission
- History of malignancy, severe obesity, and pregnancy
- History of any hospitalization within the last 3 weeks of ICU admission in the hospital
- Death or discharged from ICU against medical advice without completing the 14-day of anidulafungin therapy

### Statistical analysis and ethical approval

Data were analyzed with SPSS version 22.0 statistical software (SPSS, Chicago, IL, USA). All tests were two-sided. Pearson’s chi-square test was performed for comparing the categorical variables. Student’s t-test was used for comparing continuous variables. Values were expressed in mean ± SD (standard deviation) with 95% confidence intervals (CI). A multivariable log-binomial model was used to estimate the relative risk (RR) of odds between the groups (case and control). To analyze overall survival in the groups (EAT vs NEAT group) we plotted Kaplan-Meier curves. A *p* value <0.05 was considered as statistically significant. The research related to human use has been complied with all the relevant national regulations, institutional policies, and in accordance with the tenets of the Declaration of Helsinki, and has been approved by the Research Ethics Committee, Square Hospitals Ltd, Dhaka, Bangladesh (REC/SHL-OR-110319) on March 26, 2019. Written consent was taken from all participants in this study.

## Results

Following the inclusion and exclusion criteria strictly, total 149 (N) patients were selected for the study, where 77 and 72 patients were considered for EAT group (as case samples) and NEAT group (as control samples), respectively. All the patients of this study were South Asian population. The mean (± SD) age of EAT and NEAT group patients were 60.4 (± 17.5) and 61.3 (± 17.6), respectively (*p*<0.05), and in both the groups, number of male patient was higher than female patients (male/female: 41/36 and 40/32 in NEAT and NEAT group, respectively) (Table 1). Comorbidities in all patients of the study are descriptively mentioned in Table 1. In terms of infection markers in blood, in addition to white blood cell (WBC) count, C-reactive protein (CRP) and procalcitonin levels were per formed in all sepsis patients of case and control group in early hours of ICU admission to clearly determine the presence and severity of sepsis. Table 1 showed that the mean (± SD) CRP levels in the patients of EAT and NEAT group were 210.2 (± 112.5) and 221.9 (± 105), respectively and the *p* value was not significant. The mean (± SD) procalcitonin levels in the patients of EAT and NEAT group were 8 (± 25.9) and 10.8 (± 27.2), respectively with a *p* value >0.05 (Table I). The mean (± SD) WBC count of the patients of case group (18.3 (± 4.7)) was very close to the mean (± SD) WBC count of the patients of control group (18.1 (± 4.9)) which was statistically not significant (Table I). The serum creatinine level was determined in every patient of both the groups during admission to determine the kidney condition. The mean (± SD) serum creatinine level in patients of EAT and NEAT group were 1.7 (± 0.6) and 1.6 (± 0.6), respectively (*p* value = 0.900). Markers for lung function, cardiac function, and liver function in all patients of the study at the time of admission in ICU are mentioned in Table 1. The Acute physiology and chronic health evaluation (APACHE) II Score was estimated for every newly ICU-admitted patients of both the groups to determine the severity of illness. In the EAT and NEAT group’s patients, the estimated mean (± SD) APACHE II Score were 18.7 (± 4.4) and 17.2 (± 4.4), respectively, which was statistically significant (*p* value = 0.005).

**Table 1:** Demographic, information, comorbidities, laboratory test results during admission in patients received empirical anidulafungin or no empirical antifungal therapy

Characteristics	Variables (N = 149)		P value
	EAT group (Case) (n = 77)	NEAT group (Control) (n = 72)	
Age (year)			
Mean ± SD	60.4 ± 17.5	61.3 ± 17.6	
Range (min-max)	20 - 85	22 - 90	0.762

Gender			
Male	41	40	
Female	36	32	0.870

Comorbidities			
Diabetes, n (%)	64 (83.11)	59 (81.94)	0.001
Hypertension, n (%)	68 (88.31)	57 (79.16)	0.001
CVD, n (%)	37 (48.05)	40 (55.55)	0.842
BA, n (%)	23 (29.87)	26 (36.11)	0.577
CKD, n (%)	25 (32.46)	26 (36.11)	0.565
COPD, n (%)	14 (18.18)	12 (16.66)	0.784
CLD, n (%)	11 (14.28)	8 (11.11)	0.685
PUD, n (%)	9 (11.68)	11 (15.27)	0.723
Arthritis, n (%)	4 (5.19)	3 (4.16)	0.067
PD, n (%)	6 (7.79)	4 (5.55)	0.023

C-reactive protein (<10.0 mg/mL)			
Mean ± SD	210.2 ± 112.5	221.9 ± 105	
Range (min-max)	11.7 - 390.4	30.4 – 388.4	0.515

Procalcitonin (<0.1 ng/mL)			
Mean ± SD	8 ± 25.9	10.8 ± 27.2	
Range (min-max)	0.02 - 114	0.08 - 129	0.522

White blood cell (4-11 K/μL)			
Mean ± SD	18.3 ± 4.7	18.1 ± 4.9	0.998
Range (min-max)	12.4 - 32.6	11.5 – 30.2	

Serum creatinine (0.8-1.4 mg/dL)			
Mean ± SD	1.7 ± 0.6	1.6 ± 0.6	0.900
Range (min-max)	0.6 - 3.3	0.7 – 2.9	

SpO2 (%), Mean ± SD	92 ± 2	91 ± 3	0.586
Range (min-max)	86 - 96	87 - 98	

RSO, Mean ± SD	7 ± 6	7 ± 5	0.644
Range (min-max)	2 - 14	2 - 15	

Respiratory rate, (breaths/min), Mean ± SD	24 ± 3	25 ± 5	0.144
Range (min-max)	19 - 31	17 - 29	

Heart rate (beat/min), Mean ± SD	98 ± 14	98 ± 9	0.062
Range (min-max)	72 - 112	72 - 109	

LDH ((U/L), Mean ± SD	432 ± 189.4	467 ± 167.1	
Range (min-max)	389.4 – 526.7	376.3 – 632.4	0.048

ALT (U/L), Mean ± SD	67 ± 36.6	73 ± 41.2	0.058
Range (min-max)	59.8 – 88.3	56.2 – 92.6	

AST (U/L), Mean ± SD	39 ± 16.7	36 ± 19.3	0.264
Range (min-max)	32.4 – 59.7	33.7 – 72.3	

APACHE II Score (0-30)			
Mean ± SD	18.7 ± 4.4	17.2 ± 4.4	0.005
Range (min-max)	10 - 24	10 - 30	

SD = standard deviation; n = number; % = percentage; min = minimum; max = maximum; CVD = cardiovascular disease; BA = bronchial asthma; CKD = chronic kidney disease; COPD = chronic obstructive pulmonary disease; CLD = chronic liver disease; PUD = peptic ulcer disease; PD = parkinson’s disease; SpO_2_ = oxygen saturation in blood; min = minute; RSO = requirement of supplemental oxygen; CRP = C-reactive protein; mg = milligram; L = liter; FEU = fibrinogen equivalent units; mg = milligram; ng = nanogram; dL = deciliter; K/μL = thousand cells per micro liter; LDH = lactate dehydrogenase; U/L = units per liter; dL = deciliter; ALT = alanine aminotransferase; AST = aspartate aminotransferase; APACHE = Acute physiology and chronic health evaluation.

In the EAT group, after receiving anidulafungin empirically for 14 days, 5.19% (n = 4) of patients developed IC during their hospital stay where CP and CUTI was found in 3.8% (n = 3; *Candida albicans* in 1 patient and non-*albicans Candida* in 2 patients) and 1.2% (n = 1) of patients respectively. In contrast, 29.17% (n =21) of patients in NEAT group were experienced with IC during their hospital stay where CP was occurred in 22.2% of patients (n =16) (*Candida albicans* in 11 patients and non-*albicans Candida* in 5 patients); CUTI was detected in 4.1% of patients (n = 3) (*Candida albicans* in 2 patients and non-*albicans Candida* in 1 patient); and candidemia was found in 2.7% of patients (n = 2) (*Candida albicans* in 1 patient and non-*albicans Candida* in 1 patient). The rate of occurrence of IC with *Candida albicans and* non-*albicans Candida* in patients of NEAT group was more than 5-fold higher than that of the EAT group’s patients (29.17% versus 5.19%, respectively; *p* value <0.05) (Table 2). In comparison to the EAT group, the relative risk of occurrence of IC in the patients of NEAT group was 0.175 (95% CI, 0.0640.493; *p* value <0.05) with a risk difference (RD) rate of 24% (95% CI, 12.36%-35.58%).

**Table 2:** Incidences of invasive candidiasis and mortality rate in patients

Group	IC* occurred during hospital-stay (%)			P value	30-day mortality rate (n)	P value
	4 (5.19%)					
EAT group (n = 77)	Candida pneumonia 3 (3.8)		Candida UTI 1 (1.2)		10.39 % (8)	
	1 (CA*); 2 (NAC)		1 (NAC*)			
				<0.001		0.040
	21 (29.17%)					
NEAT group (n = 72)	Candida pneumonia 16 (22.2)	Candida UTI 3 (4.1)	Candidemia 2 (2.7)		19.44 % (14)	
	11 (CA); 5 (NAC)	2 (CA); 1 (NAC)	1 (CA); 1 (NAC)			

IC*: invasive candidiasis; CA*: *Candida albicans*; NAC*: non-*albicans Candida*

**Fig. 1:**
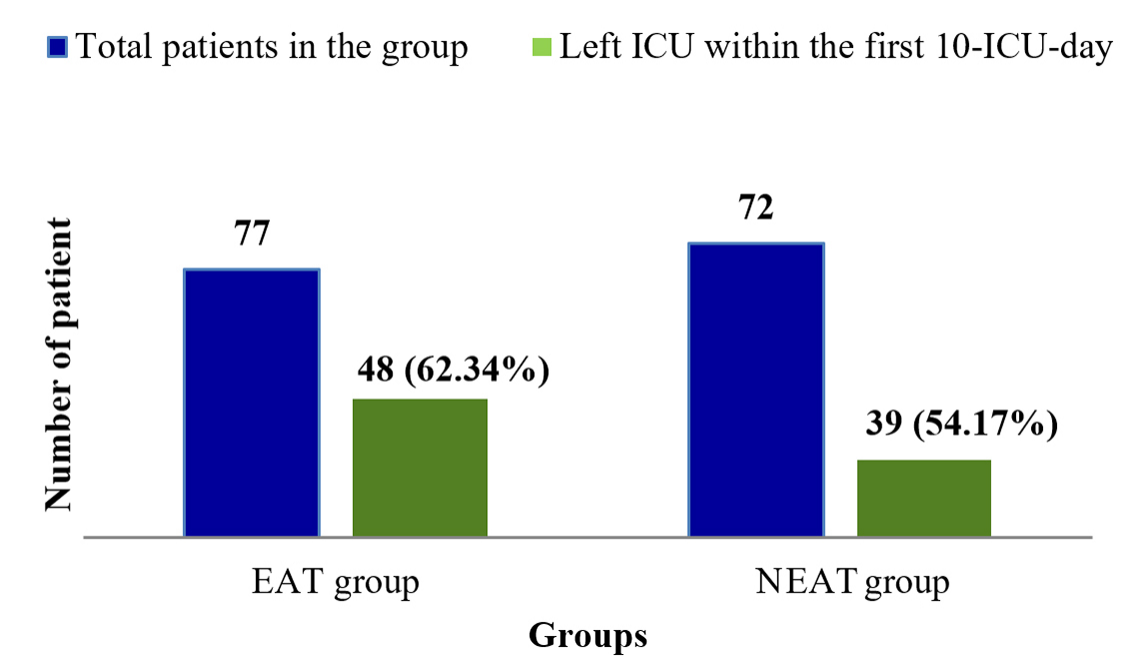
ICU leaving rate within the first 10 day of ICU admission

**Fig. 2:**
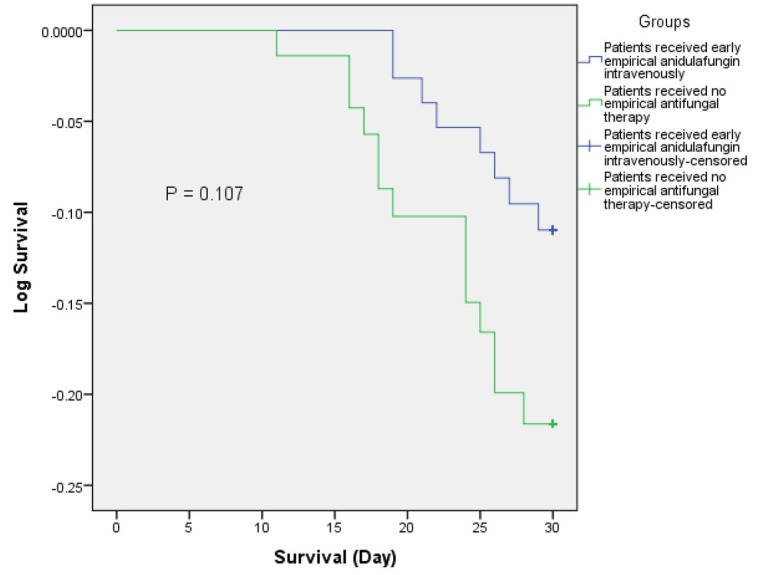
Kaplan-Meier survival curve for EAT group (received intravenous anidulafungin empirically) (blue line) and NEAT group (received no antifungal therapy empirically) (green line). Analysis run using groups (EAT vs. NEAT) as factor; death as event and time to death as time variable.

Considering all the causes of mortality during the first 30-day of hospital stay, higher mortality rate was observed in the patients of NEAT group (19.44%) than the patients of EAT group (10.39%), and that was statistically significant (*p* = 0.04). Patients leaving ICU early indicates faster recovery from the critical illness such as sepsis with other comorbidities. Patients in the EAT group showed higher ICU leaving rate (62.34%, n =48) within the first 10-ICU-day than the patients of NEAT group (54.17%, n = 39) (Figure 1). The The Kaplan-Meier 30-day survival curve was analyzed using groups (EAT vs NEAT group) and illustrated in Figure 2.

## Discussion

In this study, patients (EAT group) received empirical anidulafungin therapy within 6 h of ICU admission developed significantly less invasive candidiasis (5.19%) during their ICU stay compared to patients (NEAT group) received no empirical antifungal therapy (29.17%). The 30-day mortality rate was also less in EAT group’s patients (10.39%) than that of NEAT group’s patients (19.44%). Invasive candidiasis is always considered as a serious infection in the critically ill patients of ICU [23]. IC caused by *Candida* species is most common in ICU [1] and accountable for 8-9% of all bloodstream infections in critically ill patients [24]. With the crude hospital mortality rate of 40-54%, globally IC is now a burning question in ICU patients to treat it, and associated bacterial infections make its diagnosis and treatment more complicated [25]. Conventional treatment protocols with azoles and amphotericin B to treat IC has raised the question of increasing mortality and morbidity rate in critically ill patients, and that is why, clinicians are now looking for more effective and efficient treatment options for preventing or treating IC in ICU [11, 23]. In this consequence, all the American and European experts have recommended incorporating empirical antifungal therapy in high-risk critically ill patients, however, a promising empirical antifungal drug for IC prevention is still a matter of debate [25, 26]. Over the last decade, few studies tried to establish echinocandins empirically in the prevention of IC in critically ill patients and found potential outcomes [7, 8, 13, 22]. In 2001, a surveillance report on 10-year laboratory repots of deep-seated mycoses in England and Wales showed that *Candida* species are the most common cause of IC among the critically ill patients and accountable for 70-90% of all invasive mycoses [27]. For several decades, amphotericin B deoxycholate was used as the standard therapy for invasive fungal infections, however, because of its poor tolerability and nephrotoxicity, it has lost its acceptance. In the late 1980s, first miconazole and ketoconazole, and after that fluconazole and itraconazole came as alternatives to amphotericin B deoxycholate in the clinical practices but, fluconazole became the most popular in IC prevention and treatment [28]. A study on Prospective Antifungal Therapy (PATH) Alliance database in North America found that candidemia was caused more by non-*Candida albicans* species rather than *C. albicans*, *Candida glabrata* and *Candida krusei, and these were less susceptible to fluconazole [29]. Another epidemiological study in Spain found that among the Candida species causing candidemia frequently, Candida albicans (45.6%) and Candida parapsilosis (33.1%) were the most common in adults [13]*. Multiple randomized controlled trials (RCTs) tried to address the clinical outcomes of fluconazole in patients with complicated disease states [26]. A RCT study conducted from 1995 to 2000 on 270 adult patients with fever despite of administrating broad-spectrum antibiotics in 26 ICU patients in the United States. The study found no significant clinical outcomes with high-dose of fluconazole in comparison to placebo treatment [30]. Later on, another RCT study compared caspofungin with placebo started empirically in 222 adult mechanically ventilated patients with nosocomial sepsis receiving antibiotic therapies, simultaneously. However, no significant difference observed between caspofungin and placebo therapy in reducing the rate of IC event [31]. Another RCT study was conducted to compare the outcomes between caspofungin and placebo intended to treat IC in high risk critically ill patients, however, interrupted prematurely in 2015 because of inadequate enrollment of patients in the study [26]. Timsit and colleagues [32] conducted EMPIRICUS trial (double blind placebo controlled trial) in 23 French ICU patients. All were mechanically ventilated non-immunocompromized adult patients with sepsis of unknown origin and having at least one extradigestive fungal colonization site. The study reported that empirical micafungin therapy significantly reduced fungal-free survival up to 28^th^ day of ICU admission in comparison to the placebo therapy.

Anidulafungin, a novel broad‐spectrum antifungal, is a new member of echinocandins that has potential activity against *Candida* and *Aspergillus* species but, no activity against *C. neoformans* [33]. Nowadays, the increasing trend of resistance of *Candida* species to fluconazole and its dissatisfactory clinical outcomes with high mortality and morbidity rate discourages the clinicians to use fluconazole empirically in diagnosed or undiagnosed invasive fungal infections in critically ill patients. Therefore, the alternate option of using echinocandins including anidulafungin empirically is coming up strongly [34, 35, 36, 37]. A quantitative review of randomized trials illustrated the superiority of echinocandins in comparison to fluconazole or amphotericin B as the first-line therapy in critically-ill patients [38]. A medical ICU-based prospective cohort study found that empirical echinocandins revealed better potentiality in the reduction of IC events in critically ill patients despite IC remained unclear [7]. Similarly, our study found better clinical outcomes with early empirical anidulafungin therapy with reduced mortality rate in the patients with known sepsis or septic shock and unproven fungal infection. Several studies found a potent *in vitro* fungicidal activity of anidulafungin against a wide range of *Candida* species, including the strains resistant to fluconazole [39, 40, 41, 42]. *In vitro* data showed that anidulafungin is less potent against *Candida parapsilosis* isolates [43, 44, 45] but, it is more potent to this species than caspofungin and micafungin [46]. Animal studies and *In vitro* data demonstrate that anidulafungin is active against *C. albicans* and *C. parapsilosis* biofilms [47], and exhibits post‐antifungal effect (PAFE) (i.e. ongoing antifungal activity at limited exposure to an antifungal) like other members of echinocandins class [48]. An *in vitro* study reported that anidulafungin possesses PAFE for a longer period even at concentrations below its minimum inhibitory concentration (MIC) compared with fluconazole, caspofungin and amphotericin B, and in contrast, fluconazole has no PAFE effect at any of its concentration, caspofungin has a shorter PAFE period (0-2 h) and amphotericin B also exhibits a shorter duration of PAFE at levels below the MIC [49]. Exceptionally, some *Candida* isolates survive at even higher echinocandins’ concentrations than MICs; this is called paradoxical growth or Eagle effect. The rate of *in vitro* paradoxical growth among those *Candida* species is lower with anidulafungin than with caspofungin and micafungin, and the actual mechanism is still unknown [50, 51].

Pulmonary infections with *Candida* species in critically ill patients are associated with high rate of mortality [52]. Multiple studies demonstrated successful treatment of CP with anidulafungin alone^52^ and also in combination with intravenous voriconazole [51, 52], and maintained adequate concentration of anidulafungin in alveolar macrophages with excellent bronchopulmonary penetration ability [53]. *Candida* UTI and candidemia are also frequent in ICU patients with high rate of mortality and morbidity [1, 5]. Candidemia is accountable for 5.6 to 10% of nosocomial bloodstream infections [55, 56, 57], and studies found that anidulafungin exhibits superior clinical outcomes than fluconazole in the treatment of bloodstream infections caused by *Candida* strains susceptible or resistant to fluconazole [33, 58, 59]. In this study, *Candida* pneumonia was relatively higher than *Candida* UTI and candidemia in both groups, and patients of NEAT group demonstrated higher rate of CP (22.2%) than the patients of EAT group (3.8%). In NEAT group, among the total 16 cases of CP, 11 cases were found with *Candida albicans* where 5 cases were happened with non-albicans *Candida*. There was no evidence of candidemia in case group received anidulafungin empirically. The higher incidence rate of fungal infections (*Candida* pneumonia, *Candida* UTI and candidemia) in the patients of NEAT group (22.2%) may contributed in the increased rate of hospital mortality (19.44%) than the patients of EAT group (10.39%).

Use of broad spectrum antibiotics, steroid therapies, and immunosuppressants increases the risk of overgrowth of fungus in different parts of host body and make them virulent which results in fungal infections. Unlike bacterial infections, in-hospital secondary fungal infections are less considered by the clinicians in critically ill patients. As a result, late diagnosis delays the essential anti-fungal treatment. In this study, critically ill patients with sepsis and bacterial pneumonia received most of the aforementioned immune system modifying agents for a long-time for treating diseases which might allowed secondary opportunistic infections, such as invasive fungal infections to happen. For a confirmatory diagnosis of an invasive fungal infection without distinguishable signs and symptoms in the critically ill patients during the early hours of ICU admission is still a complicated issue for the clinicians worldwide [22]. Initiation of IC treatment based on microbial culture report is a time consumable basis of treatment, and in some cases, clinical conditions of patients are extremely deteriorated before getting the culture report in hand [58]. Study found that approximately 40%-50% of patients are admitted in ICU with candidemia and its diagnosis takes days to confirm [2, 61]. As a result, the length-of-stay in ICU and the associated treatment cost are increased [62]. This study found that empirical anidulafungin therapy potentially contributed in the overall improvement of clinical conditions of critically ill sepsis patients which ultimately resulted in increased ICU leaving rate (62.34%) of the patients of EAT group within the first 10-ICU-day of ICU admission. On the other hand, patients of NEAT group with no empirical anidulafungin showed a reduced ICU leaving rate (54.17%) within the first 10-ICU-day; but after a confirmative diagnosis of IC (based on culture-sensitivity report), patients of NEAT group received anidulafungin. In comparison to fluconazole, higher clinical outcome was attributed with anidulafungin [59,63,64] with 13.9 more hospital-free days [63]. In our study, the use of early empirical anidulafungin therapy significantly reduced the rate of occurrence of IC and 30-day mortality more in the case group’s patients (with higher ICU leaving rate within the first 10-ICU-day) than that in patients of control group received no empirical anidulafungin therapy (RR was 0.175, 95% CI, 0.064-0.493, *p* value <0.05).

This study had some limitations including small sample size; no advanced laboratory facility of the study setup to identify different *Candida* isolates from specimens; no treatment-cost analysis of patients with empirical anidulafungin therapy; and no analysis of hospital-free days. In future, some prospective studies with large sample size and some RTC studies are highly required to clearly determine the therapeutic-superiority of anidulafungin compared to fluconazole and amphotericin B in terms of clinical outcome, mortality rate, ICU leaving rate and cost-effectiveness of antifungal treatment in critically ill ICU patients in developed, middle-income, lower middle-income and low-income countries as well.

## Conclusion

This study found that critically ill patients received intravenous empirical anidulafungin within 6 h of ICU admission showed low risk of invasive candidiasis during ICU stay with reduced 30-day all-cause mortality rate and higher ICU leaving rate within the first 10-ICU-day compared to patients found no empirical anidulafungin therapy.
